# Migratory Herbivorous Waterfowl Track Satellite-Derived Green Wave Index

**DOI:** 10.1371/journal.pone.0108331

**Published:** 2014-09-23

**Authors:** Mitra Shariatinajafabadi, Tiejun Wang, Andrew K. Skidmore, Albertus G. Toxopeus, Andrea Kölzsch, Bart A. Nolet, Klaus-Michael Exo, Larry Griffin, Julia Stahl, David Cabot

**Affiliations:** 1 Faculty of Geo-Information Science and Earth Observation (ITC), University of Twente, Enschede, The Netherlands; 2 Max Planck Institute for Ornithology, Department of Migration and Immuno-Ecology, Vogelwarte Radolfzell, Radolfzell, Germany; 3 Department of Animal Ecology and Project group Movement Ecology, Netherlands Institute of Ecology (NIOO-KNAW), Wageningen, The Netherlands; 4 Institute of Avian Research, Wilhelmshaven, Germany; 5 Wildfowl & Wetlands Trust, Slimbridge, Gloucestershire, United Kingdom; 6 Sovon Dutch Centre for Field Ornithology, Nijmegen, The Netherlands; 7 Environmental Consultancy Services, Carrigskeewaun, Carrowniskey, Westport, Co. Mayo, Ireland; The Ohio State University, United States of America

## Abstract

Many migrating herbivores rely on plant biomass to fuel their life cycles and have adapted to following changes in plant quality through time. The green wave hypothesis predicts that herbivorous waterfowl will follow the wave of food availability and quality during their spring migration. However, testing this hypothesis is hampered by the large geographical range these birds cover. The satellite-derived normalized difference vegetation index (NDVI) time series is an ideal proxy indicator for the development of plant biomass and quality across a broad spatial area. A derived index, the green wave index (GWI), has been successfully used to link altitudinal and latitudinal migration of mammals to spatio-temporal variations in food quality and quantity. To date, this index has not been used to test the green wave hypothesis for individual avian herbivores. Here, we use the satellite-derived GWI to examine the green wave hypothesis with respect to GPS-tracked individual barnacle geese from three flyway populations (Russian n = 12, Svalbard n = 8, and Greenland n = 7). Data were collected over three years (2008–2010). Our results showed that the Russian and Svalbard barnacle geese followed the middle stage of the green wave (GWI 40–60%), while the Greenland geese followed an earlier stage (GWI 20–40%). Despite these differences among geese populations, the phase of vegetation greenness encountered by the GPS-tracked geese was close to the 50% GWI (i.e. the assumed date of peak nitrogen concentration), thereby implying that barnacle geese track high quality food during their spring migration. To our knowledge, this is the first time that the migration of individual avian herbivores has been successfully studied with respect to vegetation phenology using the satellite-derived GWI. Our results offer further support for the green wave hypothesis applying to long-distance migrants on a larger scale.

## Introduction

Satellite remote sensing is increasingly being used in ecological studies [1]–[4] and some new systems are facilitating the use of satellite data in ecological studies. For example, the Environmental-Data Automated Track Annotation (Env-DATA) System enables the processing of a large array of remote sensing weather and geographical data to analyze spatio-temporal patterns of animal movement tracks [5]. The integration of passive acoustic monitoring (PAM), visual sighting surveys, satellite telemetry records, and photo-identification catalogs in a biogeographic database (OBIS-SEAMAP) is another example of a system that provides new views and tools for assessing the ecology of marine mammals and biodiversity on a global scale [6].

The normalized difference vegetation index (NDVI) is a global vegetation indicator derived from remote sensors that integrate signals from the red (RED) and near-infrared (NIR) reflectance of Earth’s objects, according to the equation: NDVI = (NIRRED)/(NIR+RED) [7], [8]. NDVI calculations are based on the principle that actively growing green plants strongly absorb radiation in the visible region of the spectrum, while strongly reflecting radiation in the near-infrared region. NDVI is therefore interpreted as a measure of green leaf biomass [9]. Since the plant biomass trends generally correspond to the trend in NDVI [10] and the NDVI is closely related to net primary productivity [11], the NDVI derived from multispectral satellite data is commonly used by ecologists to estimate vegetation biomass (e.g. food quantity) as well as to assess seasonal changes in plant biomass over large regions [1], [12].

Satellite NDVI time-series data has also been widely adopted as a proxy for plant phenology in ecological studies [13]–[16]. The plant phenology itself has been recognized as a good proxy for plant quality, as young plants are generally of high nutritional value, with low levels of secondary plant chemicals [17]. The nutritional quality declines with maturation stage (or vegetative biomass) [18]. Forage quality is highest during the early phenological stages (young growing plants) and then declines rapidly as the vegetation matures over the growing season [19]. Recent studies in the Arctic tundra using plant data [20] have shown that three NDVI metrics are significantly related to the date of peak nitrogen concentration. The strongest relationship was found with the date at which NDVI values reached 50% of their annual maximum (*R* ^2^ = 0.87).

NDVI has been employed as a proxy for the forage quality and timing of the availability of high-quality vegetation in studies of herbivore behavior and habitat use. For example, Mueller et al. [21] examined the relationship between vegetation productivity and animal habitat utilization, and they found that the intermediate range of NDVI was significantly associated with the highest food quality and resource availability for herbivores like Mongolian gazelles (*Procapra gutturosa)*. Hamel et al. [22] assessed the relationship between two NDVI indices and the date of peaks in fecal crude protein, which represents temporal variability in the high-quality vegetation available for alpine ungulates. They concluded that NDVI can reliably be used to measure the yearly changes in the timing of the availability of high-quality vegetation for temperate herbivores.

Further support for the use of NDVI is provided by several more examples: Ryan et al. [23] studied the relationship between NDVI and forage nutrient indicators in a free-ranging African herbivore ecosystem. They suggested that NDVI can be used to index the nitrogen content of forage and that this is correlated with improved physical condition in African buffalo (*Syncerus caffer*). An individual-based movement modeling approach has been used to investigate how changes in NDVI, i.e. spatio-temporal variability in vegetation productivity, affected the migratory movements and their timing for zebra [24] and elephants [25]. Stoner et al. [26] used NDVI to evaluate the relative differences in habitat quality between the home ranges of natal and adult cougars (*Puma concolor*).

It has been hypothesized that movements of migratory herbivores are linked to plant phenology. This so-called green wave hypothesis states that herbivores time their spring migration to take advantge of successive peaks of nutrition and digestibility of plant growth as they migrate toward their breeding destination [27]. A space-time-time matrix of greenness is a tool for relating instantaneous green-up (or any other resource state) to animal movement [28]. It was calculated from satellite NDVI time-series data, and used by Bischof et al. [28] to study the relationship between plant phenology and the use of space by migratory and resident red deer (*Cervus elaphus*). They found that migrants had much greater access to early plant phenology than the residents. Deer were also more likely to migrate to areas that provided greater gains in instantaneous rate of green-up, which was interpreted as “springness” [28]. Rather than “surfing the green wave” during their migration, the red deer moved rapidly from the winter to the summer range, thereby “jumping the green wave”. The space-time-time matrix of greenness was also defined as the relative phenological development. It has been successfully used to explain the difference in altitudinal migration between giant pandas (*Ailuropoda melanoleuca*) and golden takin (*Budorcas taxicolor bedfordi*) in relation to spatio-temporal variations in food quality and quantity [29]; the indicator of greeness was called the satellite-derived green wave index (GWI) in our study. Although the satellite-derived GWI has been proved to be a useful tool to study the migration of herbivorous mammals with respect to vegetation phenology, it has never been tested for migrating avian herbivores. We therefore set out to investigate the satellite-derived GWI for three different populations of barnacle geese (*Branta leucopsis*).

Barnacle geese are highly selective herbivores [30], and they prefer to eat the parts of a plant with the highest nutritional quality [31]. The green wave hypothesis has been successfully tested for this species using direct field measurements of plant biomass and quality at selected field sites [19]. Moreover, the timing of the spring migration in European greater white-fronted geese (*Anser albifrons*) in relation to the green wave has been well predicted using peaks in the acceleration of temperature (GDDjerk), which seem to be closely related to the onset of spring [32].

Our aim was to test if the satellite-derived GWI can be used for studying the green wave hypothesis with respect to avian herbivore migrants. We therefore examined a prediction based on the green wave hypothesis: if barnacle geese are surfing the green wave, then the phase of vegetation greenness they encounter will closely match the 50% GWI (i.e. the assumed date of peak nitrogen concentration).

## Materials and Methods

### Study Area and Barnacle Goose Populations

**Figure 1:**
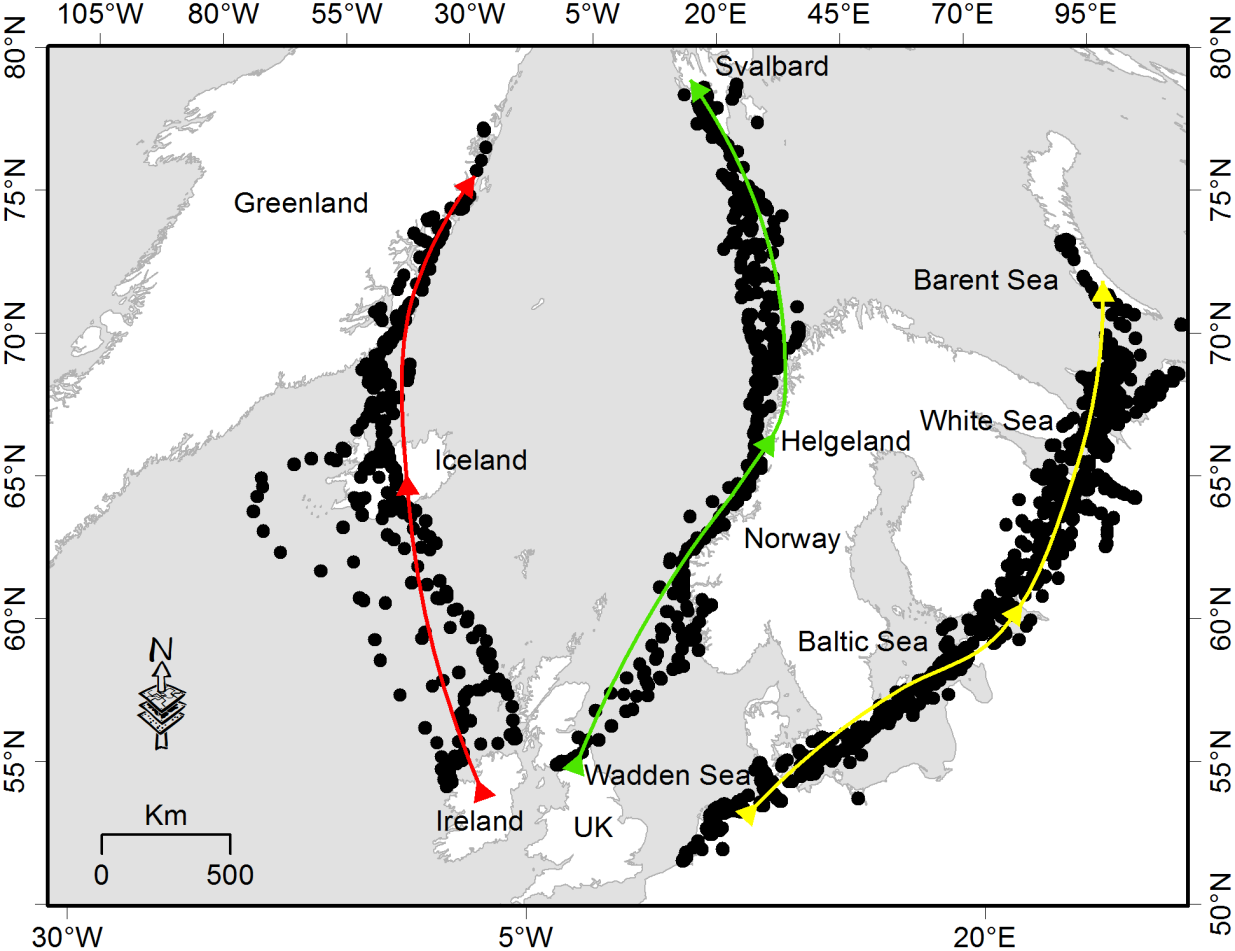
Spring migration route for three barnacle goose populations from their wintering to their breeding sites. The yellow, green and red arrows indicate the Russian, Svalbard and Greenland flyways, respectively. In each flyway, the dots show examples of the spatial distribution of GPS locations recorded for the 12 Russian, 8 Svalbard and 7 Greenland barnacle geese, from 2008 to 2010.

There are five separate populations of barnacle geese in the Western Palearctic, including three Arctic and two temperate breeders [31], [33]. We studied the three long-distance migratory populations, from Russia, Svalbard (Norway), and Greenland, which use different wintering sites but breed in the Arctic (Figure 1).

In order to catch and fix transmitters on barnacle geese, we obtained a license under the Wild Flora and Fauna Protection Act (*Flora en Fauna Wet*), number FF75A/2007/056, and approval from the Dutch Ethical Committee, under protocol number CL 0703. A license to conduct this study in the Natura2000 area “*Waddenzee*” was obtained from the Province of Friesland, number 00692701. In the UK, permission to fit satellite tags was granted by the British Trust for Ornithology Unconventional Marks Panel. The Greenland barnacle geese were caught and fitted with transmitters under a license issued by the National Parks and Wildlife Service, Dublin, under the Wildlife Act, 1976, section 32.

The Russian barnacle goose population overwinters on the Wadden Sea coast of Denmark, Germany and the Netherlands. The geese leave this area in April-May and migrate via stopovers in the Baltic Sea (most notably on the Swedish island of Gotland and in western Estonia), the White Sea, and the Kanin Peninsula. They arrive at their breeding sites on the Arctic coast of Russia in early June, after a flight of 3000–3700 km [34], [35]. The Svalbard population overwinters on the Solway Firth, UK. Birds leave from mid-April onwards, and typically have stopovers on the coastal islands of Norway (Helgeland in mid-Norway, and Vesterålen in northern Norway) for two to three weeks. They arrive at their breeding sites in Svalbard from mid-May onwards, after flying some 3100 km [31], [36]. The Greenland population leaves its overwintering sites on islands off the north and west coasts of Scotland and Ireland in the second half of April. They migrate via stopovers in Iceland and arrive at their breeding sites on northeast Greenland in late May [37].

### MODIS NDVI Data

We used the 16-day composite MODIS NDVI data (MOD13A2) (http://glovis.usgs.gov/), collected by NASA’s MODIS Terra satellite at a 1-km resolution and spanning the period from 2008 to 2010. This is useful for continental and global ecological studies [8], [38]. The MODIS NDVI product is given in the sinusoidal projection system that ensures consistency of the size of the sites, independently of their latitude. The composition methods that are used to produce the MOD13A2 products reduce artifacts due to clouds, aerosols and satellite-view zenith angle [8]. However, some noise from residual cloud and aerosol contamination, as well as sensor problems, remain in the data, which causes misclassification of phenological parameters [8]. In order to minimize the overall noise in the NDVI time series, a Savitzky-Golay filter was applied to each annual NDVI cycle. In the next step, double logistic function-fitting, suitable for modeling the yearly NDVI time series of boreal and arctic-alpine vegetation, was applied to maintain the integrity of the time series data [14], [39].

The effects of snow and large solar zenith angles at high latitudes cause a dramatic decrease in the NDVI during the winter [40]. Since snow cover negatively affects the NDVI, the melting snow at the end of winter allows the NDVI to rise, although the rise is not necessarily related to increased vegetation activity [41]. To reduce the effect of snow in high latitudes, the winter NDVI (i.e. the NDVI of any snow-affected pixel during the winter season from October until February) was therefore estimated using a method proposed by Beck et al. [14].

For our next analysis we aimed at a temporal resolution of 1 day rather than that of the 16-day composite, so the 23 NDVI images were interpolated to 365 images for each year using simple linear regression.

### Satellite-Derived Green Wave Index (GWI)

The satellite-derived green wave index (GWI) is a transformation of the interpolated NDVI and has a ratio output ranging from 0–100% for each cell and indicating the annual minimum and maximum NDVI, respectively. The greenness of two pixels at a given time can be compared by looking at the GWI irrespective of their absolute NDVI, because the GWI is normalized to account for differences such as land cover variances [13], [38]. The GWI were calculated following the method proposed by White et al. [13] and Beck et al. [38]:
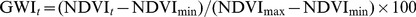
(1)where for each pixel NDVI_min_ is the annual minimum NDVI, NDVI_max_ is the annual maximum NDVI, and NDVI*_t_* and GWI*_t_* are the NDVI and green wave index at time *t*, respectively [13], [38]. The pixels with GWI = 0, or near 0%, appear in areas that are at, or near, their minimum greenness. The pixels with GWI of 100%, or near 100%, indicate areas that are at, or near, their maximum greenness [42]. A GWI of 50% indicates the intermediate stage of the greenness and incorporates a quality versus quantity trade-off (i.e. an area with high quality forage) [20], [43].

### GPS Tracking Data of Barnacle Geese

The geese were captured on their overwintering sites in the Netherlands, Solway Firth, and Ireland, and fitted with solar GPS/ARGOS transmitters (Solar GPS 100 PTT; PTT-platform transmitter terminal; Microwave Telemetry, Inc., Columbia, MD, USA). The Russian and Svalbard barnacle geese were equipped with 30 g transmitters (except for the individuals with ID 78198, 78378 and 178199 in the Svalbard population, which were equipped with 45 g transmitters). The Greenland barnacle geese were equipped with 45 g transmitters (except for the individuals with ID 65698 and 70563, which were equipped with 30 g transmitters). The PTTs were programmed to record the position of the individual goose four times per day for the Russian population, and every two hours for the Svalbard and Greenland populations, from dawn to dusk. The data collected included the goose ID, date, time, longitude, latitude, speed, course, and altitude. The GPS locations were uploaded to ARGOS satellites every four days [44]–[46]. From the Russian population, 12 females were tagged, whereas from the Svalbard and Greenland population, 15 males were tagged in total. However, the barnacle goose is a monogamous species and pair bonds persist during migration and for a long period, so the data sets were comparable [27].

For each of the three years (2008–2010), GPS tracks of incomplete spring migrations were removed from our analysis, resulting in 26 full data tracks for 12 female birds of the Russian population, 9 full data tracks for 8 male birds of the Svalbard population, and 7 full data tracks for 7 male birds of the Greenland population (see Table 1). The barnacle geese tracking data of all three populations can be viewed at movebank.org:

**Table 1:** Tag ID, year of tracking, and number of stopover sites for each barnacle goose.

Russian population (n = 12)			Svalbard population (n = 8)			Greenland population (n = 7)		
Bird ID	Track year	No. ofstopoversites	Bird ID	Trackyear	No. ofstopover sites	Bird ID	Track year	No. ofstopover sites
78033	2009–2010	2	33953	2010	2	65698	2009	2
78034	2009–2010	2	33954	2010	1	70563	2010	2
78035	2009–2010	2	78198	2008	5	78199	2010	2
78036	2009–2010	3	78378	2008–2009	3	78207	2008	2
78037	2009	2	86824	2009	1	78208	2008	2
78039	2009–2010	4	86828	2009	1	78209	2008	1
78041	2008–2010	6	178199	2008	3	78210	2008	3
78043	2008–2010	10	186827	2009	2			
78044	2008–2010	10						
78045	2008	4						
78046	2008–2009	2						
78047	2008–2010	10

- Russian population: “Migration timing in barnacle geese (Barents Sea), data from Kölzsch et al. and Shariatinajafabadi et al. 2014”, DOI:10.5441/001/1.ps244r11
- Svalbard population: “Migration timing in barnacle geese (Svalbard), data from Kölzsch et al. and Shariatinajafabadi et al. 2014”, DOI:10.5441/001/1.5k6b1364
- Greenland population: “Migration timing in barnacle geese (Greenland), data from Kölzsch et al. and Shariatinajafabadi et al. 2014”, DOI:10.5441/001/1.5d3f0664.

### Delineation of Stopover Sites

During their spring migration, the geese stop at several sites along the way to rest, refuel or await better weather conditions [36]. To delineate stopover sites for each individual, groups of continuous GPS positions were identified where the movements of individuals between two positions in a cluster were no greater than 30 km, which is the maximum distance between resting and foraging grounds at wintering sites [32]. The stopover sites were selected where the birds remained for at least 48 h in such a GPS cluster [47]. The location of each site was defined as the center of each selected group, by taking the average of the latitudes and longitudes of the GPS positions [32]. In total, for 2008 to 2010, we recognized 57 stopover sites along the Russian flyway, 18 along the Svalbard flyway, and 14 along the Greenland flyway (for 12, 8 and 7 geese, respectively) (see Table 1).

### Relating Satellite-Derived Green Wave Index to Barnacle Goose Migration

We used two approaches to test whether barnacle geese ‘surf’ along the green wave. One approach was a visualization method to identify correlations between barnacle goose movements during the spring migration and vegetation phenology. For the visualization method, first we divided the study area into three flyways, i.e. Russian, Svalbard and Greenland. Then we used the GPS-tracking data of migrating barnacle geese and related these to the spatio-temporal pattern in GWI (i.e. the vegetation phenology). In this regard, the annual GWI trajectories were stratified for each flyway separately by latitude, plotted along axes of time and latitude, and colored according to GWI value. Thus, each cell in the stratified image represented the average of the actual GWI values in each latitudinal band at a certain time.

The timing of 50% NDVI correlates with the peak in food quality [20]. So, our second approach was to define the date at which the actual GWI value reached 50% of its annual maximum at each of the stopover sites, and compare that to the date on which the geese arrived at that site using regression analysis. To perform the analysis, data from different stopover sites were combined from the three years for each population, leading to 57 stopover sites for the Russian population, 18 for the Svalbard population, and 14 for the Greenland population.

To predict the geese arrival dates from three populations at each stopover site, we used a linear, mixed-effect model, with a fixed effect for the date of 50% GWI, as well as considering the random effect of individual geese within different tracking years and the random effect of each tracking year.

A slope approximately equal to 1 and an intercept near 0 represents surfing the green wave (i.e. where the date of 50% GWI at a given stopover site was also the date on which that stopover was occupied by the geese). The coefficient of determination, *R^2^*, was used to assess the strength of the relation.

In addition to regression analysis, we calculated the root-mean-square deviation (RMSD) to measure how well the observed arrival dates at stopover sites fitted with arrival dates predicted from the satellite-derived GWI. We defined RMSD values<10 days as a good fit, 10–15 days as moderate, and >15 days as poor, based on Duriez et al. [48].

The effect of tracking year and flyway on the actual GWI values was tested using a two-way factorial ANOVA, with year (three levels) and flyways (three levels) as well as their interaction. Where a significant effect was found, we used a Bonferroni correction at *p* = 0.0167 to compare means within each factor level.

Barnacle geese forage on food patches with the highest grass density [31] and they also forage on agricultural fields in temperate regions [19], [34]. We therefore extracted the actual GWI values only from grassland and cropland land cover types in a 15-km radius around each of the 57, 18, and 14 stopover sites for the Russian, Svalbard, and Greenland populations respectively (Appendix S1). This distance is based on the core foraging range for barnacle geese [49]. In order to do the statistical analysis (i.e. regression and ANOVA), the actual GWI values were extracted from the real stopover site locations.

## Results

### Visualization of Barnacle Goose Migration against Satellite-Derived GWI

**Figure 2:**
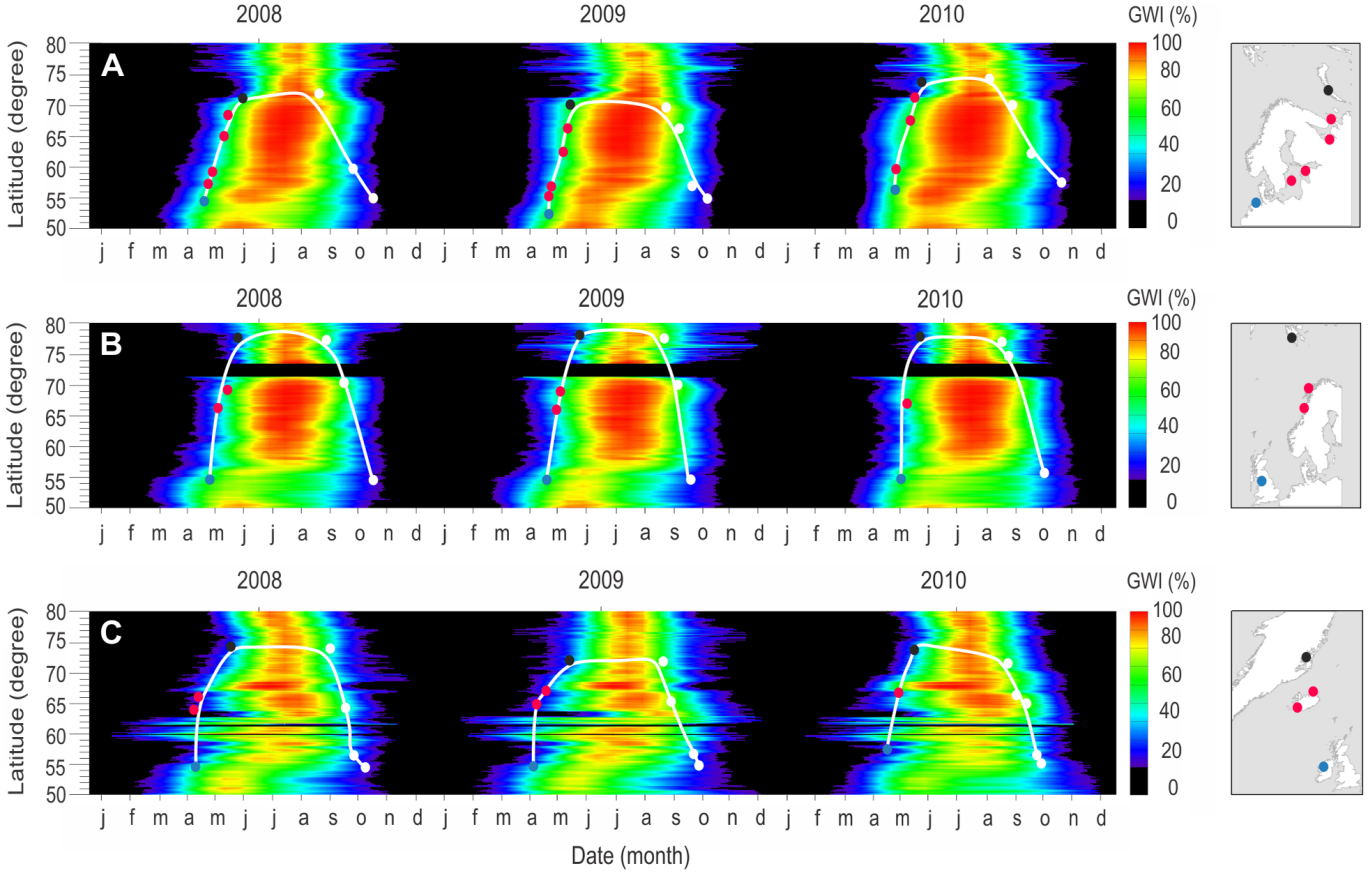
The GWI summary plots showing plant phenology over three years (2008–2010). The Russian (A), Svalbard (B) and Greenland (C) flyways are indicated. The GWI is estimated from MODIS NDVI and ranges from 0% (minimum greenness) to 100% (maximum greenness). The northward spring migration has been shown on the GWI background, as well as the return movement throughout the year. Each dot in the figure represents the average of both the latitude of the site locations and the time for 12 Russian, 8 Svalbard and 7 Greenland barnacle geese, from 2008 to 2010. The site locations include breeding (black dots), overwintering (blue dots), and stopover (red dots) sites for the spring migration and white dots for the autumn migration. The map of each flyway with the site locations overlaid is shown in the right-hand column. The white smoothed line shows the general migration pattern of the geese with respect to the vegetation phenology. The black bands on the western flyways (Svalbard and Greenland) indicate areas with no NDVI information (i.e. ocean).

The northward migration of barnacle geese correlated well with the plant phenology (Figure 2). Their spring migration during the study period fell within the early stage (GWI 20–40%), middle stage (40–60%), or late stage greenness (60–80%) based on the GWI values.

In two years, 2008 and 2009, Russian barnacle geese left the lower latitudes in late-April, when the GWI values were near to 70%. For a one-month period (late-April to late-May), the geese migrated to higher latitudes, following a mid-range of GWI values (GWI 40–60%). They arrived at the breeding sites, where the GWI values were close to 20%, at the end of May and beginning of June. The Svalbard geese followed the same phenological stage of the vegetation as the Russian geese, but stayed closer to 40% GWI during their migration to higher latitude.

In contrast, the spring migration of the Greenland geese and their response to the plant phenology was different to the other two populations. The Greenland geese left the lower latitudes around the start of April, when the GWI was about 40%. During their migration to higher latitudes, they tracked a constant but lower range of GWI values (20–40%) than the Russian and Svalbard geese, i.e. the Greenland geese followed an earlier stage of the GWI than the Russian and Svalbard geese (2008 and 2009 in Figure 2). However, in 2010, we observed that the geese from all three populations tracked a higher range of GWI during their northward migration. The GWI range was 60–80% for the Russian and Svalbard geese, whereas it was 40–60% for the Greenland geese. Indeed, in 2010, the GWI values showed that all the tracked geese migrated northward when the vegetation was in a later phenological stage than the two preceding years (Figure 2). In all three years, the maximum greenness was rarely attained for the habitats between 50–55 latitude in each of the flyways (Figure 2). Unlike the spring migration, the autumn migration of barnacle geese did not fall in a specific GWI stage but instead they followed a rather wide range of GWI (Figure 2).

**Figure 3:**
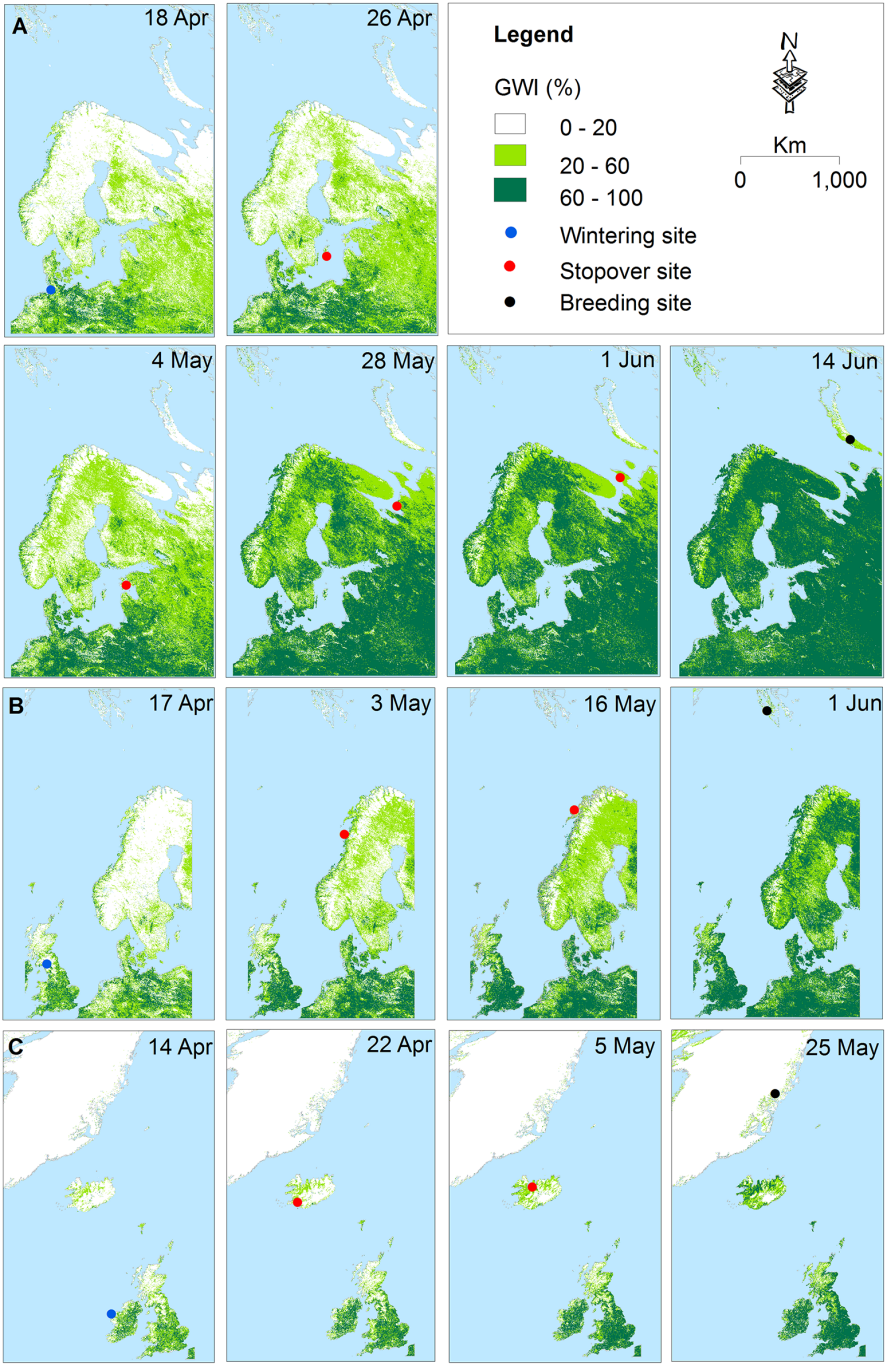
The northward movement of three individual barnacle geese in relation to the green wave. The map indicates the Russian (A), Svalbard (B), and Greenland flyways (C). The individuals’ IDs were: 78045, 178199, and 78207 for birds on the Russian, Svalbard and Greenland flyways, respectively, in 2008.

In order to further illustrate how barnacle geese follow the phenological development of the vegetation, the GWI was mapped during the spring migration in 2008 and showed the barnacle goose locations for the corresponding time periods (Figure 3). This map strongly supports the hypothesis that phenological development drives barnacle goose movement during the spring migration.

### Correlation between Barnacle Goose Spring Migration and Date of 50% GWI

For individuals from the Russian flyways, the residual variance estimate (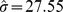) was larger than the random effect variance estimates of individual geese within different tracking years (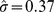) and given the random effect of a tracking year (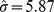). Moreover, for individuals on the Svalbard and Greenland flyways, we determined an estimate of zero for the random effect variance; this simply indicated that the level of “between-group” and “within-group” variability is not sufficient to warrant incorporating a random effect in the model. We therefore eliminated the random effect from the model and fitted an OLS regression to individuals on the Russian, Svalbard and Greenland flyways.

**Figure 4:**
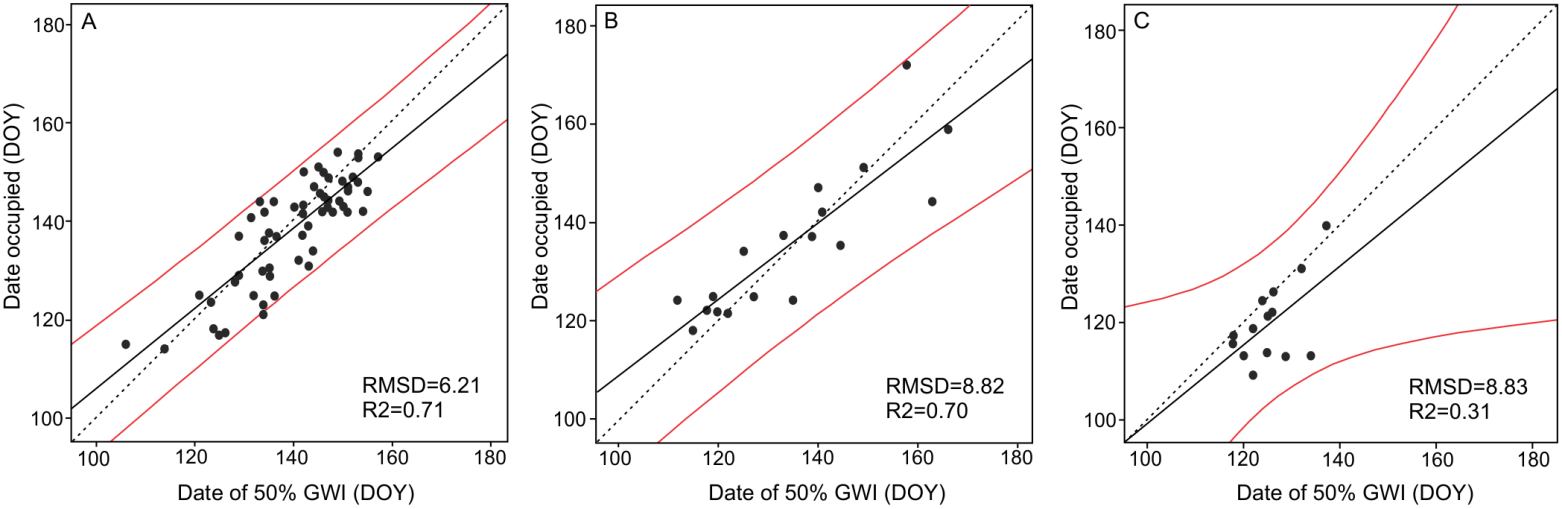
The relationship between date of 50% GWI and arrival date at stopover sites during migration. The Russian (A), Svalbard (B) and Greenland (C) barnacle goose populations are indicated. The solid black line shows the OLS regression line, while the dotted line is the 1∶1 line. The red line shows the 95% confidence interval. GWI = green wave index, DOY = day of the year counting from 1^st^ January.

In all three flyways, we found a significant relationship between the arrival dates at the stopover sites and the date of 50% GWI at that specific stopover (Table 2). However, the relationship was stronger for the Russian (*R^2^* = 0.71, *p*<0.001, *n* = 57) and Svalbard geese (*R^2^* = 0.70, *p*<0.001, *n* = 18) than for the Greenland geese (*R^2^* = 0.31, *p*<0.05, *n* = 14) (Table 2, Figure 4). Furthermore, there was a good fit between observed arrival dates at stopover sites and arrival dates predicted using the GWI index for the Russian (RMSD of 6.21), Svalbard (RMSD of 8.82) and Greenland geese (RMSD of 8.83) (Figure 4).

**Table 2:** Results of ordinary least squares regression between the arrival date of the barnacle geese at the stopover sites and the date of 50% GWI, for three different flyways, from 2008 to 2010.

Flyway	d.f.	*R^2^*	*p-*value	Coefficient	Intercept
Russia (n = 57)	55	0.71	<0.001	0.86	20.31
Svalbard (n = 18)	16	0.70	<0.001	0.90	11.96
Greenland (n = 14)	12	0.31	<0.05	0.38	79.20

d.f. degree of freedom, *R^2^* coefficient of determination.

### Comparison of GWI at Spring Stopover Sites for the Three Flyway Populations

**Figure 5:**
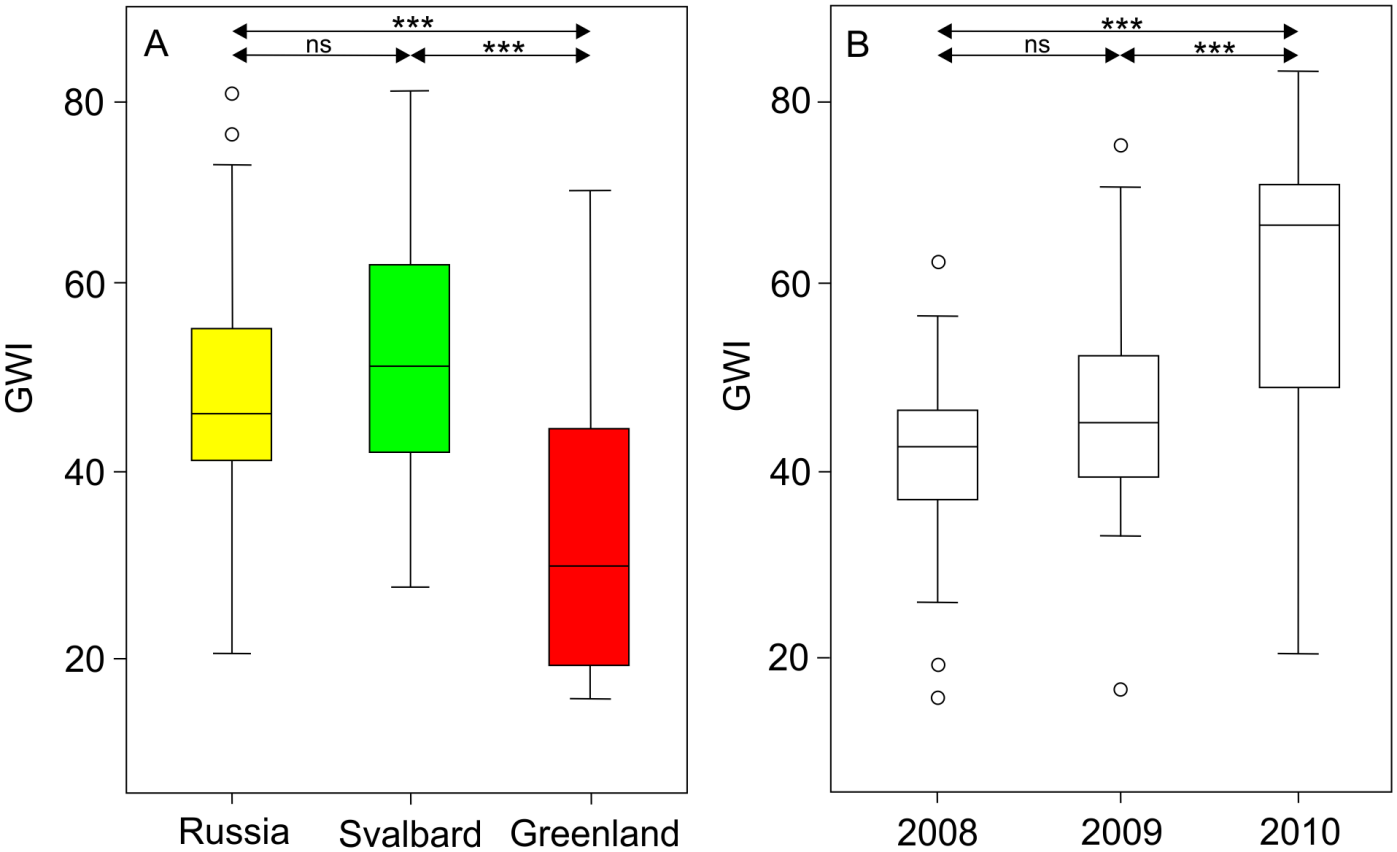
Box plots showing the development of the green wave index (GWI) at stopover sites. The range of GWI values is shown for the three flyways (A), and for the three different years (2008–2010) (B). Each box plot shows the median (line within the box), the 25th percentile (lower end of the box), the 75th percentile (upper end of the box), and 10th to 90th percentile (solid lines). The open circles show the outliers. The significant differences in GWI at the stopover sites between the three different flyways and the three different years seen in an ANOVA analysis using a Bonferroni correction are indicated (here *p-*value = 0.05/3). ***p≤0.001, ns = non-significant.

A factorial ANOVA revealed a significant main effect of flyway on GWI values at stopover sites (Table 3). It suggested that the GWI values at the stopover sites in the Russian and Svalbard flyways were significantly higher than at the stopover sites in the Greenland flyway (Figure 5A). Moreover, the GWI was affected by year and it was significantly higher in 2010 than in the other years (Table 3, Figure 5B). The difference in GWI values between the Russian and Svalbard flyways and between the years 2008 and 2009 was not significant (Figure 5A, and 5B). We could not find a significant interaction effect between the year and flyway on the GWI values at stopover sites (Table 3).

**Table 3:** Summary statistics of a factorial ANOVA examining the effects of flyway, year and their interaction on GWI values at stopover sites.

Source of variation	d.f.	*F*-value	*p-*value
Flyway	2	12.68	<0.001
Year	2	14.1	<0.001
Flyway*year	4	0.96	0.43

*p-*value<0.001, n = 89, *R^2^* = 0.44.

d.f. degree of freedom, *R^2^* coefficient of determination.

## Discussion

### Migratory Barnacle Geese Track Satellite-Derived Green Wave Index

Using the satellite-derived green wave index (GWI), we have shown how strongly the spring migration of barnacle geese is correlated with the “green wave” of vegetation phenology. To our knowledge, this is the first time that the migration of individual avian herbivores has been successfully studied with respect to vegetation phenology by using the satellite-derived GWI and GPS tracking of individual birds. Our results revealed that, over a three-year period, their arrival time at the stopover sites during their spring migration coincided well with a specific range of GWI. This range is referred to as the “green wave” and we divided it into three stages (early, middle, and late) in this study. The GWI values selected at the habitat indicate that barnacle geese do not select areas with maximum plant biomass. They preferred areas with an intermediate range of plant biomass, and thereby made a trade-off between forage quality and quantity. Areas with a low GWI (<20%), where the ingestion rate is limited, and with a high GWI (>80%), where the energy intake rate decreases because of the low nutritional value and digestibility of mature forage [21], [50], were both avoided by the barnacle geese during their spring migration. Thus, their migratory behavior was consistent with the prediction derived from the green wave hypothesis – that avian herbivores follow the successive spring flushes of plants along their northward migration route. The decrease of the GWI values from June–July onwards, and thus the lack of maximum greenness for some areas of the northern mid-latitudes is presumably due to harvesting and also to the ripening and senescence of other crops in agricultural areas [51].

As Figure 2 shows, in contrast to their spring migration, barnacle geese do not appear to follow the green wave during their autumn migration. The geese are not as tied to tracking the green wave during the autumn migration because they have other constraints, such as the need to build up as good physical condition as possible after the energy stresses of the moult period. Moreover, the timing of arrival at the destination is not important in the autumn as it is in spring. They therefore tend to remain in the Arctic and accumulate fat reserves until the autumn snow forces them to migrate southwards [52] and they wait for the best weather before departing, for example to make use of tailwinds [53]. Although the geese took rests on their southward migration, they could not refuel enough during the resting periods and still depended on the energy stores they had accumulated in the Arctic before departure [54].

For the tracked barnacle geese from the Russian and Svalbard flyway populations, we found a strong significant relationship and a good fit between the arrival date at stopover sites and the dates of 50% GWI at that specific stopover (see Figure 4 and Table 2). Moreover, data points were dispersed around the 1∶1 line, and the slope of the regression line was close 1. This suggests that the Russian and Svalbard geese were able to surf the green wave and that they benefited from having access to early vegetation phenology by closely tracking the 50% GWI. However, for the Greenland geese, we observed a relatively weak relationship between arrival date and 50% GWI. Furthermore, the dispersion of data points was mostly below the 1∶1 line. This indicates that the Greenland geese arrived earlier at the stopover sites with respect to the green wave. However, their early arrival at the stopover sites may still have an advantage even if there is a lag between their arrival time and the peak in food quality. For instance, it was found that the rate of fat deposition of geese is influenced by their knowledge and experience of feeding at the same foraging sites over several years [55]. Thus, the early arrival of the geese can reduce the competition for food by deterring other birds from occupying the same foraging sites. In addition, individuals who are unable to follow the green wave properly, and thus unable to accumulate large fat reserves, would still benefit from the opportunity to breed successfully by arriving early at the breeding sites [56]. The early arrivals would have less competition for food there, and they could occupy the best nesting sites [57]. Moreover, an early start to breeding means the goslings hatch early and benefit from the longest period of high food quality and pre-migratory fettering [52].

For selective avian herbivores, such as geese, the higher nutritional quality and digestibility of plants occurs at the start of the growing season, when there is an intermediate plant biomass [31], [32]. It has been demonstrated that there was a successive wave of nutrient biomass along the spring migration route of Russian barnacle geese [19]. Moreover, along the Russian flyway, the maximum value of nutrient biomass was also found to occur at each stopover site when it was occupied [19]. The peak of forage biomass quality for Russian barnacle geese in the Baltic Sea and Barents Sea, sampled from leaf tips, was around 20^th^ April and 20^th^ June, respectively [58]. These two periods are almost similar to the arrival time of Russian barnacle geese at the Baltic Sea and Barents Sea coast seen in our study. Thus, the most plausible explanation for the association between the 50% GWI and the observed dates of geese occupying the stopover sites is that the GWI reflects the forage quality.

Our research and that done by Van der Graaf et al. [19] led to the same conclusions for Russian barnacle geese and their following of the green wave, despite using methods with very different scales. We used satellite imagery to cover the complete geographical range without any field data, while Van der Graaf et al. [19] used only field data from a limited number of sites. It is clear that using satellite imagery, such as NASA’s MODIS NDVI data which are freely available, saves a lot of time and cost for this kind of research covering vast geographical areas. Moreover, satellite imagery is available for any time period, and makes this kind of research possible in very remote areas. The satellite-derived GWI has also been successfully used to correlate the altitudes of movements of ground animals, like giant pandas and golden takin, with phenological development of the vegetation [29], [38]. Our results show that this index can also be applied to the movement of avian herbivores that move comparatively faster and cover larger distances with respect to vegetation phenology.

### Differences in the Satellite-Derived GWI at Spring Stopover Sites

The comparison of the satellite-derived GWI values at spring stopover sites between the three flyway populations showed a significant effect for the flyway. Our results showed that the Russian and Svalbard barnacle geese are more similar in terms of how they track vegetation phenology, as there was no significant difference in the GWI values between these two flyways. On the other hand, the Greenland geese were significantly ahead of the other two flyways with respect to following the green wave.

Based on the deposition rate hypothesis, birds decided to migrate when foraging conditions start to deteriorate and staying is no longer profitable [56]. The Greenland geese probably need to leave Ireland earlier because spring occurs earlier there than on the other flyways and the grass quality is assumed to decline due to maturation. These geese have no mid-point to migrate to which would be ideal in terms of surfing the green wave; instead only Iceland is available as a stopover and they must arrive there earlier in terms of spring’s progress than they would perhaps choose under more ideal circumstances.

Besides the flyways, our results showed the significant effect of the type of year on how barnacle geese follow the green wave. The Russian and Svalbard geese followed the middle stage of the green wave in 2008 and 2009, but a later stage in 2010. In contrast, the Greenland geese followed the earlier stage of the green wave in 2008 and 2009, but the middle stage in 2010. In other words, the geese we tracked followed the markedly higher value of the satellite-derived GWI in 2010 in all three populations. We think this was due to the extreme weather in northern and western Europe in 2010. The continental temperate climate zone in western Europe was particularly dry for the spring season of 2010, certainly compared with the two previous years [59]. This could have led to an earlier start to the growing season at higher latitudes because an increase in the mean annual air temperature in early spring corresponds to an advance in leafing [60]. An earlier start to the growing season at higher latitudes would have meant that the geese were more likely to catch the later phenological stages of plant growth along their flyway in 2010 if they had started migrating at their normal time. As shown by Tombre et al. [16], if the geese cannot predict the conditions they might encounter at the next stopover, they are unable to respond quickly to the advancing spring. For instance, the lack of correlation in the onset of spring between the Solway Firth and Helgeland stopovers meant the geese were unable to migrate earlier if spring was early at both sites [16]. Moreover, the timing of the Russian geese migration from the Baltic Sea was not linked to the advancement of plant growth, most likely because of the low correlation in the weather patterns between the Baltic Sea and White Sea [33]. Using the third derivative of daily temperature sums (GDDjerk), Kölzsch et al. [61] showed that the geese are able to closely follow the green wave during their spring migration if predictability of climatic conditions was high between stopovers. Therefore, in the case that predictability is low, the geese might rely more on fixed cues such as photoperiod (length of daylight hours), and do not migrate earlier in the year if spring is early.

## Conclusions

By using the satellite-derived green wave index, we have shown that individual barnacle geese surf the wave of high-nutrition plants. Remote sensing tools provide the opportunity to predict plant biomass and to study plant phenology in remote areas such as the Arctic, where it is difficult to collect plant data on a large spatial and temporal scale. In addition, by applying GWI (a metric derived from the NDVI time series) as a remote sensing tool to determine accurately the timing of high quality vegetation for herbivores (i.e. the date at which GWI reaches 50% of its maximum value), we were able to investigate how the geese from the three populations made use of the green wave during the three years studied. Remote sensing data, and NDVI in particular, are among the technological advances that are proving useful in studying large-scale movement ecology, and they have helped us gain a better understanding of how vegetation dynamics and distribution affect movement patterns in animal populations. To our knowledge, this is the first time that the migration of individual avian herbivores has been successfully studied with respect to vegetation phenology by using the satellite-derived green wave index.

## Supporting Information

Appendix S1 **The extracted GWI values from stopover sites.**
(CSV)
